# Modeling and Positioning of a PZT Precision Drive System

**DOI:** 10.3390/s17112577

**Published:** 2017-11-08

**Authors:** Che Liu, Yanling Guo

**Affiliations:** College of Mechanical and Electrical Engineering, Northeast Forestry University, Harbin 150040, China; sympic@nefu.edu.cn

**Keywords:** precision positioning, piezoelectric ceramics, PID control, creep compensation

## Abstract

The fact that piezoelectric ceramic transducer (PZT) precision drive systems in 3D printing are faced with nonlinear problems with respect to positioning, such as hysteresis and creep, has had an extremely negative impact on the precision of laser focusing systems. To eliminate the impact of PZT nonlinearity during precision drive movement, mathematical modeling and theoretical analyses of each module comprising the system were carried out in this study, a micro-displacement measurement circuit based on Position Sensitive Detector (PSD) is constructed, followed by the establishment of system closed-loop control and creep control models. An XL-80 laser interferometer (Renishaw, Wotton-under-Edge, UK) was used to measure the performance of the precision drive system, showing that system modeling and control algorithms were correct, with the requirements for precision positioning of the drive system satisfied.

## 1. Introduction

As technology advances with time, studies in various disciplines are also making progress, during which people have turned attention from macro-machinery to micro-machines. When developing micro systems, people are faced with various problems caused by high-precision instruments to realize high-precision operation. Currently, it is required that operations in the micro technique field reach nanoscale positioning that features positioning, driving, and control capacity with 0.1–100 nm precision. With vast potential for future development, nanoscale positioning systems can be used in various industrial fields, including robotics, aerospace engineering, precision machinery, rapid prototyping, automation, and ultra-precision machining.

Piezoelectric ceramic transducer (PZT) delivers no heat, noiselessness, and high positioning precision in nanoscale positioning; thus, it has been widely applied in precision manufacturing and aerospace engineering [1]. To overcome the shortcomings of PZT actuators, such as slow transient response speed, repeatability decline, and worsening controllability that are caused by the inherent characteristics of PZTs, such as creep, hysteresis, and small bandwidth [2], and to fully utilize its advantages, scholars at home and abroad have invested considerably into studying PZTs [3].

Richter et al. shortened the regulation time, improved system performance, and shortened the time when the whole platform reached stability by 3–4 ms by improving the rise time of the system step response [4]. As a model-based control, open-loop control requires mathematical models for each module in the open-loop process. Japanese scholars Seung-Woo Kim and Seung-Bae Jung developed a reference model-based open-loop control method [5].

Domestic researchers have also made outstanding contributions to PZT-based precision drive systems. Zhang Yulin, a professor from Shandong University, established an online identification model based on an artificial neural network and constructed a PZT control console based on neural network algorithms [6]. Researchers of Institute of Optics and Electronics, Chinese Academy of Sciences and Key Laboratory on Adaptive Optics, Chinese Academy of Sciences improved the dynamic positioning precision of the piezoe-lectric ceramic positioning stage based on traditional PID algorithm, the feed-forward control is constituted with the modified PI inverse hysteresis model and a traditional PID, and the experiments on slow speed and high speed positionings are performed [7]. The researchers of Southeast University propose the dynamic model and dynamic inverse model of a piezoceramic actuator. They incorporate the dynamic inverse compensation in a closed-loop PID controller to control the piezoceramic actuator. The maximum absolute error with the inverse compensation is less than 0.8 μm and that with the inverse compensation and PID is less than 40 nm in an amplitude range of 200 μm [8].

## 2. Composition of Precision Positioning Drive System 

**Figure 1:**
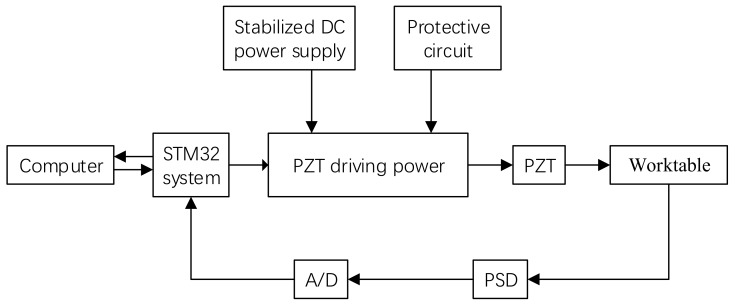
Diagram of precision positioning system.

The PZT precision drive system in this study mainly consisted of a piezoelectric ceramic transducer, drive unit, an STM32F103 single-chip computer (STMicroelectronics, Seoul, Korea Confederation), an analog/digital (A/D) conversion module, a digital/analog (D/A) conversion module, a voltage amplification circuit, and a Position Sensitive Detector(PSD) photoelectric displacement sensor, as shown in Figure 1. The STM32F103 single-chip computer is mainly responsible for logic operation control and algorithm implementation in the control system, and the voltage amplification circuit for proportional amplification, the PSD photoelectric displacement sensor for closed-loop feedback, and the PZT are used as the drive elements of the precision drive system.

### 2.1. Composition and Structure of Driving Power

**Figure 2:**
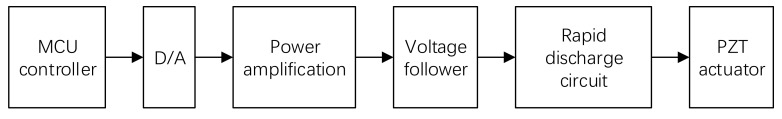
PZT driving power supply.

In PZT driving power, high-precision D/A conversion is used for output; a microcontroller is used to output digital signals that undergo D/A conversion; the converted voltage goes through power amplification to output the control voltage of the PZT, as shown in Figure 2.

### 2.2. Displacement Detection System

**Figure 3:**
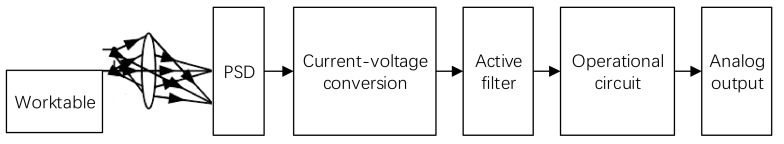
Schematic diagram of the PSD displacement detection system.

**Figure 4:**
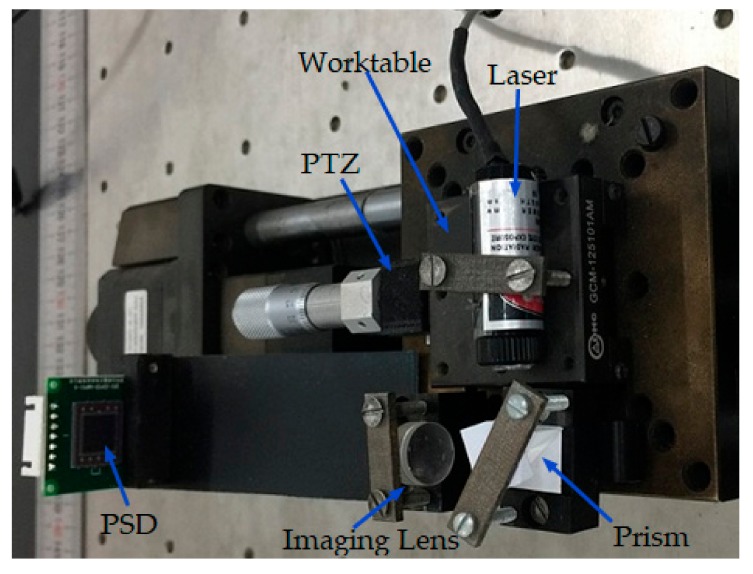
Photograph of the PSD displacement detection system.

The PSD detection system mainly consists of a micro-displacement detection device, i.e., PSD, and the following I-V conversion circuit, filter circuit, and digital operational circuit, as shown in Figure 3 and Figure 4.

In this study, a light source was fixed on the micro-control panel and moved with the worktable, which was driven by the PZT actuator. As the position of the light source changed, the voltage output by the PSD also varied accordingly, and the electrical signal was then sent to the control system through the A/D. In this study, an optical lever principle was used to amplify the micro-displacement of the PZT to improve system resolution to enable PSD positioning, ensuring its ability to reach a satisfactory level. In the center area of the PSD is approximately linear. When the light spot is away from the center, the PSD becomes nonlinear. Therefore, the center should be used as far as possible when the range is satisfied.

Here, *x′* is the actual displacement of PZT; *U*_1_ and *U*_2_ are the output voltages by the PSD; 2*L* is the length of PSD; and *β* is the magnification of the optical system.

(1) $$x^{\prime }=\frac{U_{2}-U_{1}}{U_{2}+U_{1}}\frac{L}{\beta }$$

## 3. System Modeling and Control Algorithm

### 3.1. Control System Modeling

To better analyze the system, mathematical modeling was required for the precision elements, PZT, and driving power of the control system.

**Figure 5:**
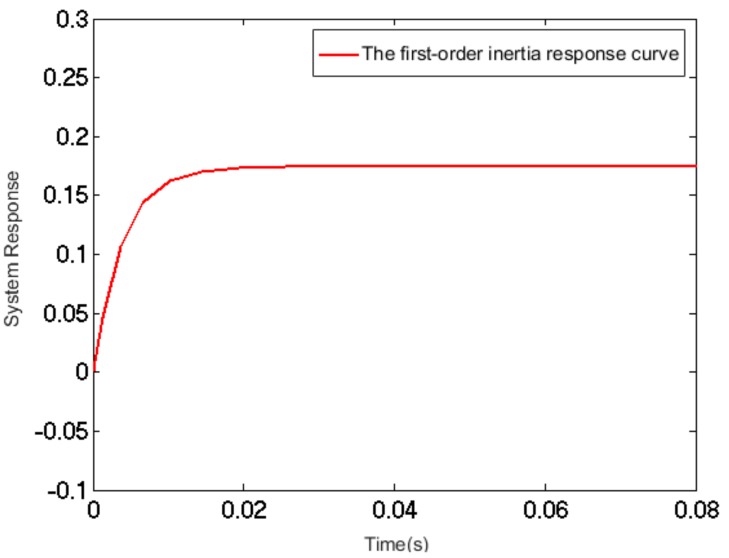
The first-order inertia response curve of PZT mathematical model.

The PZT micro-actuator can be considered equivalent to a capacitor in the circuit. With a stack structure, the equivalent capacitance of a PZT can reach as high as several microfarads. When a step voltage is applied to both ends of the PZT, it shows some features that are similar to capacitance charge-discharge. Therefore, a transient process is needed for the PZT to reach a stable state. The voltage applied is linearly correlated with the output displacement of the PZT to some degree. The PZT and power-driven equivalent resistance constitute an RC loop that is a first-order inertial loop in automatic control, as shown in Figure 5:

(2) $$G_{1}\left(s\right)=\frac{∆x\left(s\right)}{∆U_{1}\left(s\right)}=\frac{k_{m}}{T_{m}s+1},$$

where Δ*x* is the variation of displacement (μm); Δ*U*_1_ is the voltage (V) variation; *k_m_* is the displacement-voltage conversion coefficient; *T_m_* is the time constant; and *T_m_* = *R_C_* *C.*

Measured by the precision capacitance meter, the equivalent capacitance of the PZT is $C=1.96\times 10^{-6}$ µF. In consideration of the complexity of the driving power circuit, its output impedance was not a theoretical value; instead, it was measured throughout the experiment, during which an external slide rheostat was used to adjust the value of resistance, with the voltage at both ends measured according to the calculation $R_{c}^{}$ = 199.98. Thus, the time constant $T_{m}$ = $3.92\times 10^{-4}$. The PZT displacement-voltage conversion coefficient was determined by multiple metering of the PZT displacement, i.e., $k_{m}$ = 0.175:

(3) $$G_{1}\left(s\right)=\frac{0.175}{0.0039s+1},$$

**Figure 6:**
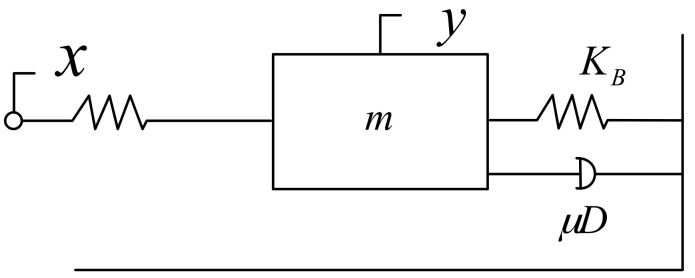
Equivalent model of the PZT displacement platform.

The PZT precision worktable could be deemed as a mass-spring-damper second-order system based on its own structure and features, as shown in Figure 6:

Here, *m* is the mass of the platform, *μD* is the damper, and *K_B_* is the elastic coefficient of the spring. According to the reference manual of the platform and relevant tests, *m* = 0.5 kg, *K_B_* = 0.83 × 10^6^ N/m, and *μD* = 249.2 Ns/m. Force analysis of the spring-mass-damping system suggested that the displacement output by the platform is *y*(*t*) in the presence of force *f*(*t*). According to Newton’s second law, there is an acceleration speed on the platform in the presence of force. The force on the platform is given as follows:

(4) $$m\frac{d^{2}y}{dt^{2}}=f\left(t\right)-f_{a}-f_{k},$$

where $f_{a}=\mu \frac{dy}{dt}$ is the damping force; $f_{k}=kx+f_{k0}$ is the spring’s resilience; and $f_{k0}$ is the resilience when the displacement is zero. With this formula, the following formula could be derived:

(5) $$m\frac{d^{2}y}{dt^{2}}=f\left(t\right)-\mu \frac{dy}{dt}-kx,$$

After Laplace transformation of this formula, we obtain:

(6) $$\frac{Y\left(s\right)}{F\left(s\right)}=\frac{1}{ms^{2}+\mu s+k},$$

**Figure 7:**
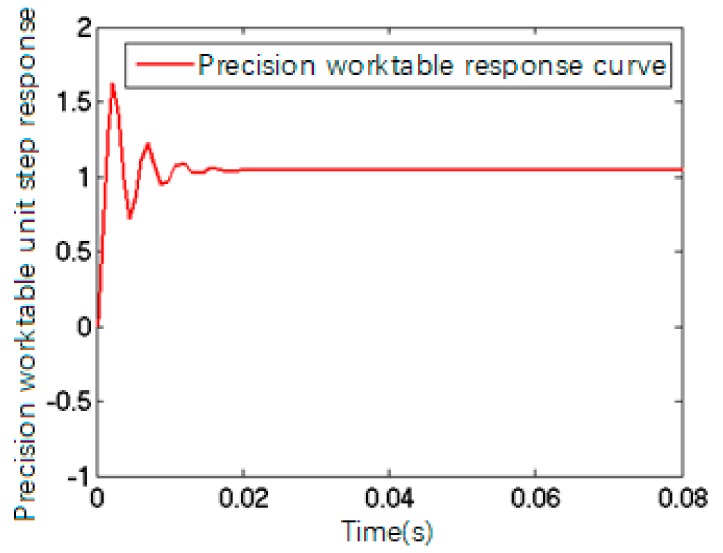
The response curve of PZT mathematical model.

When the formula above was converted into a standard second-order oscillation loop, its step response output could be described by Figure 7. Its transfer function with micron output is:

(7) $$G_{2}\left(s\right)=\frac{k_{s}\omega_{n}^{2}}{s^{2}+2\xi \omega_{n}+\omega_{n}^{2}}=\frac{2\times 10^{6}}{s^{2}+498.4s+1.66\times 10^{6}},$$

where $\omega_{n}$ is the undamped natural angular frequency of the system; $k_{s}$ is the amplification coefficient; ξ is the damping ratio.

Digital signals that needed to be converted were sent to the D/A converter through the interface circuit by the single chip computer and then converted into corresponding DC voltage analog signals. In the high voltage amplifier, low voltage output by D/A was converted into a high voltage available for PZT operation in order to control the PZT’s displacement and, thus, realize the platform positioning. The DC high voltage amplification circuit was able to amplify the analog voltage signal output by a D/A converter, which was simplified into a proportional amplifier loop under automatic control. As a result, its transfer function could be determined:

(8) $$G_{s}\left(s\right)=k_{v},$$

The amplification coefficient $k_{v}$ was consistent with the multiples of an actual operational amplifier. In this test, it was required to convert 0–5 V into 0–60 V. Therefore, $k_{v}=12$.

The proportional amplifier loop, the first-order RC inertial loop, and the second-order oscillation loop in the PZT precision worktable were connected serially, i.e., the output of the last loop served as the input of the next loop. The transfer function could be established by multiplication of the transfer functions of the various loops. The transfer coefficients of the first-order and the second-order systems suggested that the transfer function of the system is:

(9) $$G\left(s\right)=\frac{k\omega_{n}^{2}}{\left(1+T_{m}s\right)\left(s^{2}+2\xi \omega_{n}+\omega_{n}^{2}\right)}=\frac{4.2\times 10^{6}}{\left(0.0039s+1\right)\left(s^{2}+498.4s+1.66\times 10^{6}\right)},$$

Here, $k=k_{v}k_{m}k_{s}$. The open-loop transfer function of the PZT precision worktable was a third-order system whose response could be deemed as the combined action of two parts, i.e., components of the first-order inertial loop response and the second-order oscillation loop response.

**Figure 8:**
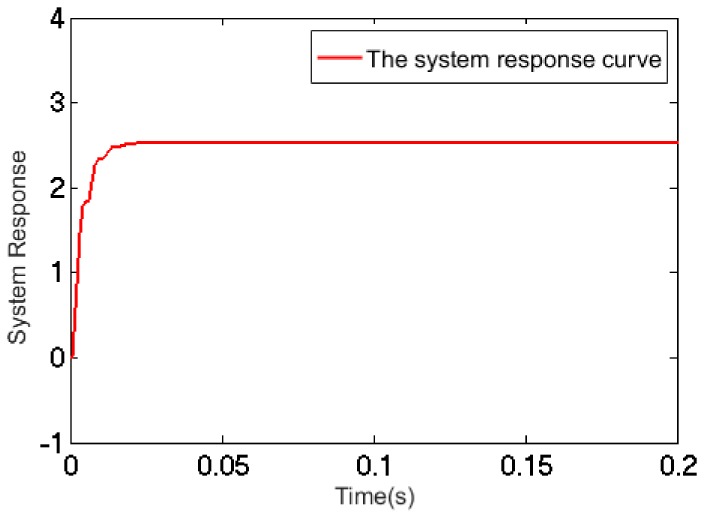
The system response curve.

The system response curve depicting the effect of a step signal is shown in Figure 8.

### 3.2. Algorithm of the Control System

The impact of creep, one of the inherent characteristics of PZTs, on PZT positioning precision could not be neglected. When a step voltage was applied to the PZT actuator, an instantaneous step response that was generally several milliseconds was generated within the time scale determined by its mechanical resonance, followed by a slow creep response. It was generally assumed that the creep process of PZT took on a logarithmic form:

(10) $$L\left(t\right)=L_{0}\left[1+\gamma log_{10}\left(\frac{t}{t_{0}}\right)\right],$$

where L(t) is the total displacement of the PZT with a given voltage, $L_{0}$ is the PZT displacement with a given voltage in time $t_{0}$, γ is the PZT creep coefficient, $t_{0}$ is the step response time, which is generally $t_{0}=0.1\;s$, *t* is the creep time, and $t_{0}$ is the zero timing point of *t*.

When voltage was applied $t_{0}=0.1\;s$, the PZT started the creep process, with the creep rate γ varying with the voltage difference and with the voltage course.

It could be assumed that it was the input voltage that led to a certain creep displacement of PZT according to the contravariant relationship between electric energy and mechanical energy, whereas constant strain could also result in the voltage creep of the PZT. Therefore, voltage creep could be analyzed by the law of displacement creep. The voltage creep model is given as:

(11) $$U\left(t\right)=U_{0}\left[1+\gamma_{𝑣}log_{10}\left(\frac{t}{t_{0}}\right)\right],$$

where U(t) is the input voltage at time *t*, $U_{0}$ is the input voltage corresponding to constant strain $L_{0}$, $\gamma_{𝑣}$ and is the voltage creep coefficient.

$U_{0}$, $\gamma_{𝑣}$, and $t_{0}$ were determined by various factors, such as PZT materials, experiment conditions, driving voltage and velocity, etc. As differences of piezoelectric materials, PZT aging, and experiment condition variations made it difficult for precise determination of the three model parameters in PZT creep formula, it is advisable to use the least square method.

**Figure 9:**
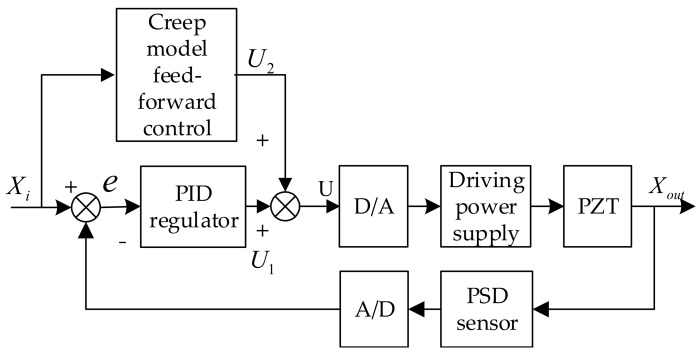
PID closed loop control chart based on feedforward control.

A creep model-based feed-forward control based on a proportional–integral–derivative controller (PID) closed-loop control was able to further reduce the impact of creep on displacement. Figure 9 shows the structure of a voltage creep model-based closed-loop control system:

In this study, a PSD feedback-based PID algorithm implementation was designed. $X_{i}$ in the control block diagram was the displacement required to be output with a certain voltage; $X_{out}$ was the output displacement of the micro-displacement platform; the deviation value between the voltage value generated by the PSD feedback displacement and input voltage was e; the voltage value u was obtained from deviation e after scale, integral, and differential operations by the PID regulator. Next, this voltage value underwent D/A where it was converted into a low-voltage control signal that was then amplified into the driving power of PZT particles, so that PZT deforms to drive the precision worktable. Displacement output by the micro-displacement platform was converted into voltage signals by the PSD sensor and was then sent to a single chip computer via an A/D converter.

Deviation e was the voltage value obtained from PSD feedback subtracting the voltage value corresponding to the displacement that was required to be output. In the event that this value lay in the deviation area, the PID control process was completed; otherwise, PID control was repeated until it fell within the designated range, i.e., the precision requirement was satisfied.

Controller tuning, also known as “optimal tuning”, is essentially matching its characteristics and controlled characteristics by adjusting the controller’s parameters, so that the PID controller is able to perform effectively. The controller parameters obtained with this method are designated “optimal tuning parameters”. Of all tuning methods, Ziegler-Nichols tuning and the performance index setting are the most widely used. Manual tuning of PID parameters using the add-up method was implemented in this test. Given that $K_{D}=K_{i}=0$, $K_{P}$ increased until there was system oscillation, then the value of this critical state was denoted as $K_{cr}$ and oscillation period as $T_{cr}$, as shown in Table 1.

**Figure 10:**
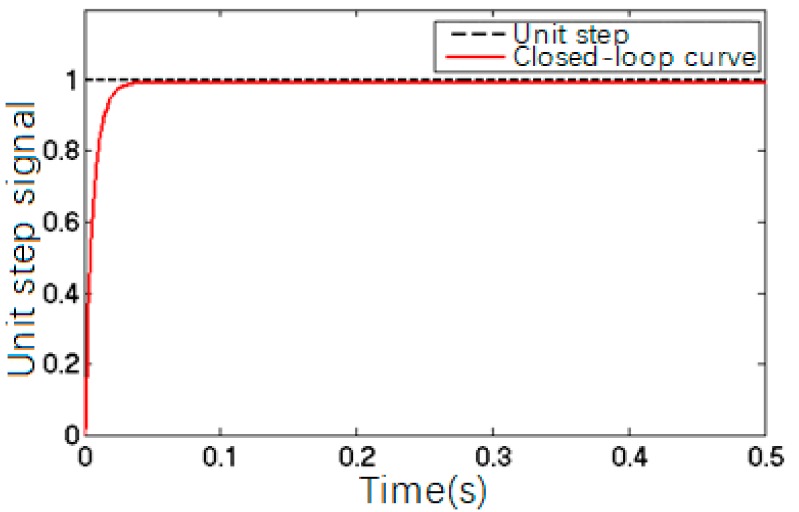
The simulation curve of the piezoelectric ceramic pressure closed-loop control.

**Figure 11:**
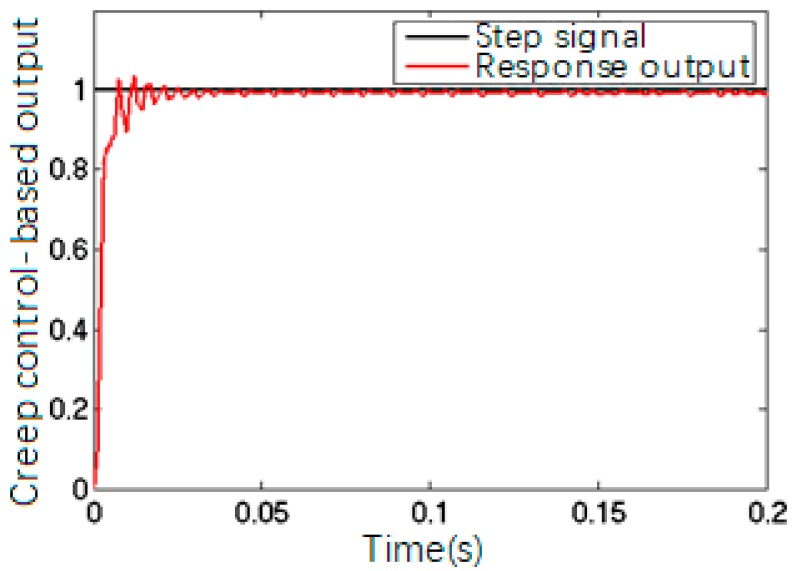
Output of the closed-loop control based on creep curve.

PID parameters obtained by Ziegler-Nichols tuning method were *K_P_* = 7.8, *K_i_* = 0.0029 and *K_D_* = 0.000725. Figure 10 shows the closed-loop response of the precision worktable with these PID parameters; Figure 11 shows the creep-based system closed-loop response.

The comparison between Figure 10 and Figure 11 suggested that the closed-loop control of a precision control system was able to trace and execute the input and output effectively with precise positioning, but the system still showed creep features, which could be solved by adding a feed-forward model to the closed-loop algorithm to eliminate the impact of PZT creep.

## 4. Test Results and Analyses

In this study PZT is the stacked type, with a size of 5 × 5 × 18 mm. The power supply of PZT is a 0–100 V adjustable DC stabilized voltage supply. The displacement was measured with the XL-80 laser interferometer from Renishaw, Wotton-under-Edge, UK. The linear measurement displacement resolution of this laser interferometer is 1 nm.

**Figure 12:**
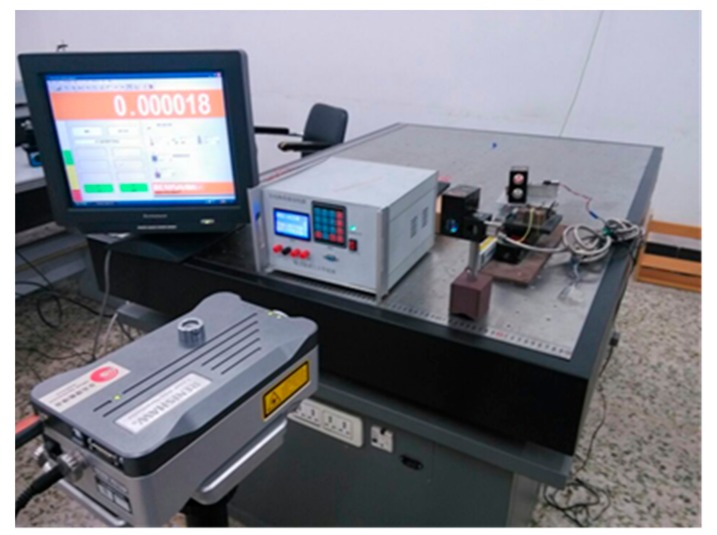
Experimental equipment of the piezoelectric ceramic control system.

Owing to the fact that the PZT showed excellent linearity after 40 V, 40 V was set to be the initial voltage. The mechanical amplification factor of the PSD photoelectric displacement sensor detection device was set to 10. The experimental system is shown in Figure 12.

**Figure 13:**
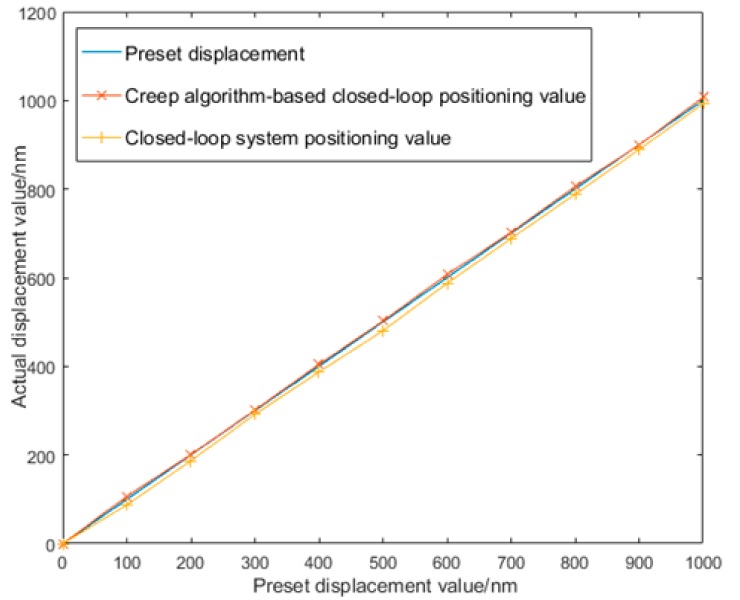
The positioning precision displacement of two algorithms under the console.

The laser interferometer was used to measure the precision worktable 20 times based on preset displacement, with the mean value taken. Table 2 and Table 3 present the measured data of the precision worktable. Figure 13 shows the curve with the preset displacement on the *x*-axis and the displacement of the closed-loop precision worktable with creep on the *y*-axis.

Closed-loop system-based positioning data was fitted to the curve *y =* 1.0001*x −* 10.966, whose linearity was 0.197% and maximum error was 19.65 nm, which was calibrated as 20 nm in the test. With creep-based feed-forward modeling, the fitted linear equation of the measured data was *y* = 0.9999*x* + 3.3668, whose linearity was 0.073% and maximum error was 7.25 nm, which was calibrated as 10 nm, given in Table 4.

When closed-loop control was used in the system, the maximum displacement error of the precision drive system could reach 19.65 nm, which was attributed to three factors. First, the overall performance of the PZT drive power, including stability and precision, would make a difference. Second, errors might also occur in the control system as a whole during installation and debugging. Most importantly, the PZT itself was not able to eliminate the impact of nonlinear features, such as creep and hysteresis, without a creep compensation algorithm. When the system was connected to a creep compensation algorithm-based closed-loop control system, the maximum error of the PZT displacement was 7.25 nm; i.e., the error caused by creep was reduced. The circuit was provided with a circuit module for quick PZT discharge, which was used to reduce the response time of the PZT when the voltage at both ends changed, thus weakening the impact of the creep features and hysteresis and improving overall system performance. Data analysis indicated that the system error with only closed-loop control was much larger than that based on the creep compensation algorithm, which possessed an absolute advantage in system performance.

## 5. Conclusions

In this study, a PID closed-loop control algorithm was the core of precision drive system, and a creep-forward compensation model based on a PSD photoelectric position feedback system was introduced. This effectively eliminates the effect of nonlinear hysteresis on PZT control accuracy and system error in control systems. The PSD detection system is compact and simple, which can be used in a limited space.

Through theoretical analysis and experiment, the algorithm is verified, and a precision drive system was designed. The maximum error of this drive system was less than 10 nm, which satisfied the requirements for precise positioning, with a high practical value in high-precision positioning systems.

## Figures and Tables

**Table 1:** Controller parameter tuning table.

Type of Controller	*K_P_*	*K_i_*	*K_D_*
P	0.5*K_cr_*		
PI	0.45*K_cr_*	0.85*K_cr_*	
PID	0.6*K_cr_*	0.5*K_cr_*	0.125*K_cr_*

P is a proportional controller, PI is a proportional-integral controller, PID is a proportional–integral–derivative controller.

**Table 2:** The measured data of the preset voltage.

Preset Voltage Value (V)	Theoretical Value (nm)	Creep Algorithm-Based Closed-Loop Positioning Value Measured by Laser Interferometer (nm)	Actual Voltage Value (V)
40.0	0	0	40.006
40.5	69	73.30	40.589
41.0	138	138.60	41.102
41.5	207	207.85	41.698
42.0	276	279.00	42.201
42.5	345	346.45	42.712
43.0	414	418.70	43.229
43.5	483	484.45	43.779
44.0	552	555.40	44.345
44.5	621	620.35	44.830
45.0	690	694.15	45.254

**Table 3:** The measured data of the preset displacement.

Preset Displacement Value (nm)	Closed-Loop System Positioning Value Measured by Laser Interferometer (nm)	Creep Algorithm-Based Closed-Loop Positioning Value Measured by Laser Interferometer (nm)
0	0	0
100	87.40	106.70
200	187.05	200.90
300	291.55	301.35
400	387.25	404.35
500	480.35	502.10
600	587.10	607.25
700	688.25	702.10
800	788.20	805.60
900	889.25	899.35
1000	992.50	1007.05
5000	4991.35	5002.45
10,000	9990.00	10,001.95

**Table 4:** Precision stage performance.

System Performance	Linearity %	Max Error (nm)
Closed loop	0.197	19.65
Addition of creep	0.073	7.25
